# Current Status of Targeted Therapy for Biliary Tract Cancer in the Era of Precision Medicine

**DOI:** 10.3390/cancers16050879

**Published:** 2024-02-22

**Authors:** Takafumi Mie, Takashi Sasaki, Takeshi Okamoto, Takaaki Furukawa, Tsuyoshi Takeda, Akiyoshi Kasuga, Masato Ozaka, Naoki Sasahira

**Affiliations:** Department of Hepato-Biliary-Pancreatic Medicine, Cancer Institute Hospital, Japanese Foundation for Cancer Research, Tokyo 135-8550, Japan; takafumi.mie@jfcr.or.jp (T.M.); takeshi.okamoto@jfcr.or.jp (T.O.);

**Keywords:** cholangiocarcinoma, biliary tract cancer, targeted therapy, precision medicine

## Abstract

**Simple Summary:**

Biliary tract cancer (BTC) is a heterogenous group consisting of intrahepatic cholangiocarcinoma, perihilar cholangiocarcinoma, distal cholangiocarcinoma, and gallbladder cancer. First-line chemotherapy in advanced BTC has been established, but evidence on second-line chemotherapy remains sparse. Advances in comprehensive genomic analysis have revealed various specific driver genes depending on BTC subtype, and nearly half of BTC cases harbor targetable genetic alterations. Here, we summarize the current knowledge on precision medicine for advanced BTC.

**Abstract:**

First-line chemotherapy has been established for advanced biliary tract cancer (BTC). However, few treatment options are available as second-line treatment. Advances in comprehensive genomic analysis revealed that nearly half of patients with BTC harbor targetable genetic alterations such as fibroblast growth factor receptor (FGFR), isocitrate dehydrogenase (IDH), BRAF, human epidermal growth factor receptor 2 (HER2), microsatellite instability (MSI)-high, neurotrophic tropomyosin receptor kinase (NTRK), rearranged during transfection (RET), and poly (adenosine diphosphate-ribose) polymerase (PARP). This review summarizes currently available options in precision medicine and clinical trials for patients with advanced BTC.

## 1. Introduction

Biliary tract cancer (BTC) is a heterogenous group consisting of intrahepatic cholangiocarcinoma (iCCA), perihilar cholangiocarcinoma (pCCA), distal cholangiocarcinoma (dCCA), and gallbladder cancer (GBC) [1]. According to the database of World Health Organization and Pan American Health Organization, the mortality rate of iCCA is increasing, while that of dCCA is declining worldwide [2]. Worldwide mortality from GBC had been decreasing over the past several decades, but has bottomed out or has even begun increase in many countries [3].

Systemic chemotherapy is the standard treatment for unresectable BTC. However, clinical trials have failed to take the heterogeneity of BTC into account. Gemcitabine plus cisplatin achieved significant survival advantage over gemcitabine monotherapy in ABC-02 trial and became the standard of care for first-line treatment in unresectable BTC [4]. In Japan, gemcitabine plus S-1 and gemcitabine, cisplatin plus S-1 triplet therapy have shown efficacy and are also considered options for first-line treatment [5,6]. Nanoliposomal irinotecan plus fluorouracil and leucovorin recently showed efficacy comparable to gemcitabine and cisplatin in a randomized phase II trial [7]. The superiority of triplet therapy consisting of gemcitabine plus cisplatin (GC) and immune checkpoint inhibitors durvalumab or pembrolizumab was shown in the TOPAZ-1 and KEYNOTE-966 trials, respectively, heralding a new era of immunotherapy in BTC [8,9]. The median overall survival (OS) achieved 12.7–12.8 months with triplet therapy consisting of GC and immune checkpoint inhibitors. In both studies, subgroup analysis showed a benefit of immune check inhibitor for median OS of the patients with iCCA, but there was no difference with extrahepatic cholangiocarcinoma and GBC [8,9]. With respect to second-line treatment, FOLFOX achieved superior overall survival relative to active symptom control alone [10]. Nanoliposomal irinotecan plus fluorouracil and leucovorin for second-line treatment after gemcitabine and cisplatin achieved better PFS than fluorouracil and leucovorin in NIFTY trial [11]. However, options for the treatment of BTC remain limited.

A key issue surrounding all clinical trials to date is the use of uniform treatment regiments despite the heterogeneity of BTC. Advances in comprehensive genomic analysis revealed that BTC have different specific driver genes depending on the BTC subtype, and 40–56% of BTC cases harbored targetable genetic alterations [12,13]. There are chemical-based and antibody-based therapies in molecular targeted therapy. In this review, we explore the targetable genetic alteration and molecular targeted therapy in BTC.

## 2. Genetic Alterations in BTC

Several studies on genetic alterations in BTC had been conducted. In their analysis of 260 Japanese patients, Nakamura et al. identified 32 significantly altered driver genes and found that nearly 40% of cases harbored targetable genetic alterations [12]. They found frequent alterations of *FGFR1*, *FGFR2*, *IDH1*, *IDH2*, *BAP1*, and *ARID1A* in iCCA, PKA and HER2 in dCCA, and EGFR, HER2, and *ERBB3* in GBC.

Jusakul et al. studied the etiopathogenetic diversity of BTC through 489 BTC patients from 10 countries [14]. *ERBB2* amplification and *TP53* mutation were frequently detected in patients with endemic liver fluke infection, while high copy-number alterations, PD-1/PD-L2 expression, epigenetic mutations (*IDH1/2* and *BAP1*), and *FGFR/PRKA*-related gene rearrangements were frequently detected in patients without endemic liver fluke infection.

Wardell et al. analyzed 412 Japanese and Italian patients [15]. MUC17 was identified as a new driver gene of BTC. Deleterious germline mutations such as *BRCA1/2*, *MLH1*, and *MSH2* were detected in 11% of cases.

Jalve et al. analyzed 554 BTC patients (412 with iCCA, 57 with pCCA or dCCA, and 85 with GBC), finding that *FGFR* and *IDH* mutations were detected exclusively in iCCA [16]. In iCCA patients, *FGFR2* mutation was more common in younger and women patients with a tendency for better prognosis, while *TP53* and *KRAS* mutations were associated with poor prognosis. Targeted therapy on genetic alterations improved prognosis in iCCA patients.

Lin et al. analyzed 803 Chinese patients and reported that *TP53*, *KRAS*, *ARID1A*, *LRP1B*, and *CDK2A* were frequently detected as cancer-related gene alterations [17]. *TP53* and *KRAS* mutations and amplified 7q31.2 were associated with poor prognosis. Targetable genetic alteration was detected in 25.4% of patients.

Umemoto et al. analyzed 3031 BTC patients, excluding iCCA patients [18]. They reported that *ERBB2* amplification was highly detected in patients with high tumor mutation burden, and *CDK12* rearrangement in patients with *ERBB2* amplification. In patients under 40 years old, *GATA6* amplification and rearrangements of *BRAF* and *FGFR2* were observed.

Li et al. analyzed 57 GBC patients [19]. *TP53*, *ERBB3*, and *KRAS* were frequently observed driver gene alterations. ErbB signaling pathway (including *EGFR*, *ERBB2*, *ERBB3*, *ERBB4*, and their downstream genes) mutations were detected in 36.8% of GBC patients and were associated with poor prognosis.

## 3. Targeted Therapy and Treatment Outcomes

Various genetic alterations have been reported in BTC as described above, some of which are druggable targets. Outcomes for BTC patients who received therapy to their actionable alteration have been reported to be favorable [13]. Major molecular-targeted agents are summarized below (chemical-based therapy in Section 3.1, Section 3.2, Section 3.3, Section 3.4, Section 3.5, Section 3.6, Section 3.7 and Section 3.8, and antibody-based therapy in Section 3.9 and Section 3.10).

### 3.1. Fibroblast Growth Factor Receptor (FGFR) Inhibitors

Four tyrosine kinase receptors, FGFR1–4, have been identified as members of the FGFR family. Binding of fibroblast growth factors to these transmembrane tyrosine kinase receptors activates downstream signaling pathways such as the JAK-STAT, RAS-BRAF-MEK-ERK, and PI3K-AKT-mTOR pathways. *FGFR2* rearrangements are considered to be one of the most critical driver genes in iCCA, occurring in 7.4–13.6% of cases [20,21,22]. They are also found in 3.6% of pCCA cases [20]. Maruki et al. reported that younger patients under 65 years old and Hepatitis C virus antibody positivity and/or Hepatitis B virus antigen positivity were associated with *FGFR2* rearrangement [20].

Pemigatinib is an oral inhibitor targeting FGFR1–3. Three cohorts were compared in a multicenter phase II study (FIGHT-202 trial): patients with *FGFR2* fusions or rearrangements, patients with other *FGF/FGFR* alterations, and patients with no *FGF/FGFR* alterations [23]. All patients included in this study had been treated with at least one chemotherapy regimen. Among patients with *FGFR2* fusions or rearrangements, pemigatinib demonstrated an overall response rate (ORR) of 35.5%, median progression-free survival (PFS) of 6.9 months, and median OS of 21.1 months.

Infigratinib is another oral inhibitor targeting FGFR1–3. A multicenter phase II study was conducted on BTC patients with disease progression during prior chemotherapy [24]. Most of the 61 patients exhibited *FGFR2* alterations (48 with *FGFR2* fusion, 8 with mutation, and 3 with amplification). Among patients with *FGFR2* fusion, the study reported an ORR of 18.8% and a disease control rate (DCR) of 83.3%. Partial response was only observed in patients with *FGFR2* fusion. Another multicenter phase II study (n = 108) on the efficacy of infigratinib was conducted in BTC patients with *FGFR2* fusion or rearrangement, revealing an ORR of 23.1%, median PFS of 7.3 months, and median OS of 12.2 months [25].

Pemigatinib and infigratinib are both ATP-competitive inhibitors which bind reversibly to ATP-binding pockets in the FGFR kinase domain. Acquired resistance mutations have been identified as potential mutations to the efficacy of these agents [26,27]. Futibatinib, on the other hand, is an oral covalent irreversible pan-inhibitor targeting FGFR1–4 with expected efficacy against acquired resistance mutations associated with ATP-competitive FGFR inhibitors. A multinational phase II study involving 103 BTC patients with *FGFR2* fusion or rearrangement after disease progression during prior chemotherapy (excluding FGFR inhibitors). This study showed an ORR of 41.7%, median PFS of 9.0 months, and median OS of 17.1 months [28].

RLY-4008, the first highly selective and irreversible oral FGFR2 inhibitor, was developed to overcome FGFR2 resistance mutations [29]. The ReFocus trial studied 38 BTC patients with *FGFR2* fusion or rearrangement who had no history of FGFR inhibitor use [30]. This trial reported a promising results, with an ORR of 88.2% and a DCR of 100%.

Derazantinib is an oral pan-FGFR inhibitor competing with ATP. In a multicenter phase I/II study involving 29 unresectable iCCA patients with *FGFR2* fusion, the reported outcomes included an ORR of 20.7%, a DCR of 82.8%, and median PFS of 5.7 months [31]. Median OS was not reached during a median follow-up period of 20 months.

Erdafitinib is an oral pan-FGFR tyrosine kinase inhibitor, exhibiting IC_50_ values in the low nanomolar range for all FGFR family constituents (FGFR1–4) [32]. In a multicenter phase I study evaluating the efficacy of erdafitinib for advanced or refractory solid tumors, 11 BTC patients with *FGFR* alterations were included (1 patient with amplification, 3 with mutation, and 8 with fusion) [33]. The study reported an ORR of 27%, and a DCR of 54%. Among all three responders of BTC, the median duration of response was 11.4 months.

KIN-3248, an oral selective, irreversible, small molecule pan-FGFR inhibitor, is being evaluated in an ongoing multicenter phase I trial for patients with advanced and metastatic solid tumors harboring *FGFR2* and/or *FGFR3* alterations [34].

Tinengotinib is an oral multiple kinase inhibitor which strongly inhibits Aurora A/B, FGFR1–3, vascular endothelial growth factor (VEGFR), and JAK1/2. A phase Ib/II study of tinegotinib for patients with advanced or metastatic solid tumors was conducted [35]. Fifteen BTC patients were included, and the ORR was 20% in BTC patients.

Major all-grade adverse events (AEs) of FGFR inhibitors included hyperphosphatemia (60–85%), stomatitis (20–37%), fatigue (25–36%), diarrhea (16–34%), nausea (12–45%), dry mouth (18–45%), dry eye (13–21%), palmar-plantar erythrodysesthesia syndrome (11–21%), and increased aspartate or alanine aminotransferase levels (20–38%) [23,24,28,30,31,33,35]. Severe AEs included hyperphosphatemia (7–30%), stomatitis (5–7%), diarrhea (3–4%), palmar-plantar erythrodysesthesia syndrome (4–5%), fatigue (1–6%), and increased aspartate or alanine aminotransferase levels (7–12%) [23,24,28]. Hyperphosphatemia is an AE unique to FGFR inhibitors. Fibroblast growth factor-23 typically regulates phosphate levels by reducing renal phosphate reabsorption and inhibiting phosphate absorption in the intestines. The inhibition of fibroblast growth factor-23 by FGFR inhibitors leads to hyperphosphatemia by increasing phosphate reabsorption in the kidney and absorption in the intestines. Hyperphosphatemia is reversible and generally controllable with a low-phosphate diet, concomitant phosphate binders, diuretics, and dose reduction or interruption [23].

### 3.2. Isocitrate Dehydrogenase (IDH) Inhibitors

*IDH* is an enzyme that converts isocitrate to α-ketoglutarate within the citric acid cycle. Mutant *IDH1* and *IDH2* convert α-ketoglutarate to 2-hydroxyglutarate (2-HG), a carcinogenic metabolite. The accumulation of 2-HG induces epigenetic changes, impairs DNA repair, disrupts cellular metabolism, and fosters tumorigenesis. The frequency of *IDH1* mutations is higher than *IDH2* mutations. *IDH1/2* mutation is observed in 20–28.8% of iCCA patients [16,36,37].

Ivosidenib is an oral, small-molecular inhibitor targeting mutated *IDH1*. In a multicenter double-blind randomized placebo-controlled phase III study (ClarIDHy trial), 185 *IDH1*-mutant patients with disease progression during prior chemotherapy were enrolled [38]. Longer PFS was achieved with ivosidenib compared to placebo (2.7 vs. 1.4 months, *p* < 0.0001). There was no difference in median OS between groups (10.3 vs. 7.5 months, *p* = 0.09). The lack of a significant difference in OS may have been due to patients in the placebo group being allowed to receive ivosidenib after tumor progression. Median OS in the placebo group was 5.1 months after adjustment for crossover, which was significantly different from that of the ivosidenib group (*p* < 0.01) [39].

Common AEs of ivosidenib included nausea, diarrhea, and fatigue. Ascites, anemia, increased serum bilirubin level, and hyponatremia were reported as severe AEs [39].

### 3.3. RAS-BRAF-MEK-ERK Pathway Inhibitors

*KRAS* mutation is a major genetic alteration that is found in 11–43% of BTC patients [12,14,16,17,18,40,41]. *KRAS* mutation is less common in iCCA than in pCCA and dCCA [16,41]. The phase II KRISTAL-1 study was conducted to evaluate the efficacy of adagrasib, an oral *KRAS G12C* inhibitor, in patients with advanced solid tumors harboring *KRAS G12C* mutations [42]. Among the 57 enrolled patients, the 12 patients with BTC showed an ORR of 41.7%, a DCR of 91.7%, median PFS of 8.6 months, and median OS of 15.1 months. This study reported AEs such as nausea, diarrhea, fatigue, vomiting, and QT prolongation.

*BRAF* encodes the B-RAF protein in the RAF family of serine-threonine protein kinases. This protein functions as the initiating enzyme in the RAF-MEK-ERK pathway. Upregulation of this pathway is associated with tumorigenesis. *BRAF* mutations are primarily observed in iCCA, found in 3.1–7.4% of cases [43,44,45]. All mutations were detected as *BRAF V600E* substitutions and associated with shorter OS [45]. The ROAR (Rare Oncology Agnostic Research) trial is a multicenter, open-label, and phase II basket trial designed to assess the efficacy and safety of dabrafenib (oral *BRAF* inhibitor) plus trametinib (oral *MEK* inhibitor) for patients for rare cancers harboring *BRAF V600E* mutations. Cases with BTC achieved an ORR of 47% based on assessment by independent reviewers, median PFS of 9 months, and median OS of 14 months [46]. Reported AEs included pyrexia, nausea/vomiting, fatigue, and rash.

Vemurafenib is an oral *BRAF V600*-mutant selective RAF inhibitor. A multicenter basket trial on 172 patients with non-melanoma *BRAF V600*-mutant solid tumors was conducted, 99% of which had *BRAF V600E* mutations and nine of which had BTC [47]. The study reported an ORR of 33.3% in BTC patients.

### 3.4. Human Epidermal Growth Factor Receptor 2 (HER2) Inhibitors

The *ERBB2* gene encodes for the protein HER2, which is a tyrosine kinase family receptor. HER2 overexpression and amplification is detected in 7.9–26.5% [48,49,50]. The frequency of HER2 overexpression and amplification with BTC patients differs among BTC subtypes, occurring less commonly in iCCA than in other subtypes [48].

The MyPathway trial is a multicenter, phase IIa, basket trial for patients with advanced solid tumors harboring targetable molecular alterations [51]. Patients with BTC harboring HER2 amplification and/or overexpression were treated with anti-HER2 humanized antibodies pertuzumab and trastuzumab, which were administered intravenously. Thirty-nine patients were enrolled in the BTC part of this study, of which the most common was GBC (16 patients, 41%). The study reported an ORR of 23.1%, median PFS of 4.0 months, and median OS of 10.9 months.

Trastuzumab deruxtecan (T-Dxd) is a novel intravenous antibody-drug conjugate comprised an anti-HER2 antibody and a cytotoxic topoisomerase I inhibitor [52]. In a multicenter, phase II study (the HERB trial), the efficacy of T-Dxd for patients with HER2-expressing advanced BTC who were refractory or intolerant to gemcitabine containing regimen was evaluated [53]. A total of 32 patients were enrolled, of whom 22 patients were HER2-positive. HER2-positive patients achieved an ORR of 36.4%, median PFS of 4.4 months, and median OS of 7.1 months. The efficacy of T-Dxd for the patients with HER2-expressing locally advanced or metastatic disease who received more than one systemic treatment or had no alternative treatments was evaluated in another multicenter, phase II study [54]. Forty-one BTC patients were enrolled, and the ORR was 22.0%. Sixteen patients (39%) with positive HER2 immunohistochemistry (IHC) status (3+) achieved an ORR of 56.3%. Median PFS and OS of all BTC patients was 4.6 months and 7.0 months, respectively, while the median PFS and OS of patients with IHC 3+ was 7.4 months and 12.4 months, respectively. Patients with IHC 3+ also showed more favorable results with T-Dxd than those with IHC 2+.

Tucatinib is an oral tyrosine kinase inhibitor highly selective for HER2. An open-label phase II basket trial that evaluated tucatinib and trastuzumab in 30 metastatic BTC patients harboring HER2 alterations (SGNTUC-019) reported an ORR of 46.7%, a DCR of 76.7%, median PFS of 5.5 months, and median OS of 15.5 months [55].

There are some reports of adding trastuzumab to existing cytotoxic chemotherapy for BTC. The efficacy of FOLFOX as second-line treatment for advanced BTC was established in the ABC-06 trial [10]. A multicenter phase II trial evaluated trastuzumab in combination with FOLFOX for BTC patients harboring HER2 alterations refractory to GC [56]. Thirty-four patients were enrolled, and an ORR of 29.4%, a DCR of 79.4%, median PFS of 5.1 months, and median OS of 10.7 months were achieved. A multicenter phase II trial was conducted to evaluate the efficacy and safety of GC plus trastuzumab as first-line treatment for BTC patients with advanced HER2-positive (defined as IHC 3+ or IHC 2+ and fluorescence in situ hybridization-positive) [57]. Ninety patients were enrolled, of whom 96% had GBC. This study reported an ORR of 55.5%, a DCR of 80%, median PFS of 7 months, and median OS of 9.96 months. Patients with isolated *TP53* mutations had worse PFS compared with patients with other mutations or with no detected mutations.

Zanidatamab is an intravenous bispecific antibody targeting two distinct HER2 epitopes. A multicenter phase IIb trial was conducted to evaluate zanidatamab for advanced BTC patients with HER2 amplification after progression on previous gemcitabine-based treatment [58]. Eighty-seven patients were enrolled, and the ORR was 41.3%, the DCR was 68.8%, the median PFS was 5.5 months, and median OS was immature as of the cutoff date.

Neratinib is an oral, irreversible pan-HER tyrosine kinase inhibitor. A multicenter, phase II, basket trial to evaluate the efficacy of neratinib for patients with solid tumors harboring HER2 somatic mutations was conducted, and outcomes of the BTC cohort have been reported [59]. Twenty-five patients were enrolled: 11 with cholangiocarcinoma, 10 with GBC, and 4 with ampullary cancer. Reported outcomes included an ORR of 16%, a DCR of 24%, median PFS of 2.8 months, and median OS of 5.4 months. Ampullary tumors and those with coexisting oncogenic *TP53* and *CDKN2A* alterations exhibited worse outcomes. This report concluded that combination therapy was preferable to improve outcomes. Major AEs of HER2 inhibitor included neutropenia, leukopenia, anemia, nausea, vomiting, fatigue, interstitial lung disease (ILD), pyrexia, diarrhea, cholangitis, decreased appetite, and increased aminotransferase [53,54,55,56,57,58]. Of note, ILD was a characteristic AE in T-Dxd and occurred in 10.5–25.0% of patients, and 1.5–12.5% experienced severe ILD [53,54].

### 3.5. Neurotrophic Tropomyosin Receptor Kinase (NTRK) Inhibitors

The *NTRK* gene family includes *NTRK1–3*. *NTRK* fusion is known to be an oncogenic driver gene in various types of tumors. Two oral *NTRK* fusion inhibitors, entrectinib and larotrectinib, are currently available. A phase I/II trial of entrectinib for patients with advanced *NTRK* fusion-positive solid tumors was conducted [60]. Fifty-four patients were enrolled. The ORR was 57%, with complete response observed in 7%. Median PFS was 11.2 months. The one included patient with BTC achieved partial response with entrectinib.

Larotrectinib was evaluated in a phase I/II trial for patients with advanced *NTRK* fusion-positive solid tumors with prior standard therapy [61]. One hundred and fifty-nine patients were enrolled. The ORR was 79%, with complete response observed in 16%. Median PFS was 28.3 months, and median OS was 44.4 months. Of the two patients of BTC, one achieved partial response, while the other had progressive disease.

These agents are promising treatment options for BTC patients with *NTRK* fusion, despite *NTRK* fusion being reported in only 0.2–0.7% of patients with BTC [62,63].

### 3.6. Rearranged during Transfection (RET) Inhibitors

The *RET* proto-oncogene encodes a transmembrane receptor tyrosine kinase. *RET* alterations induce oncogenic transformation in multiple cancers [64].

The ARROW trial was designed to evaluate efficacy and safety of pralsetinib, an oral selective *RET* inhibitor, in patients with advanced solid tumors harboring *RET* alterations [65]. In the entire cohort, the ORR was 57%, median PFS was 7 months, and median OS was 14 months. Three patients with BTC were included, of which two achieved partial response, and the third showed stable disease with some tumor shrinkage. One patient who had experienced disease progression on all three previous treatments (gemcitabine/cisplatin/abraxane, erlotinib/bevacizumab, and osimertinib), achieved a partial response with pralsetinib.

Selpercainib is another oral selective *RET* inhibitor evaluated in the LIBRETTO-001 trial, a phase I/II, open-label, basket trial, for patients with *RET* fusion-positive solid tumors excluding lung or thyroid tumors [66]. Results included an ORR of 43.9%, median PFS of 13.2 months, and median OS of 18.0 months. The sole patient with BTC achieved partial response. The most frequently observed severe AEs in this study were hypertension and increased liver enzymes.

### 3.7. Multikinase Inhibitors

Regorafenib is an oral multikinase inhibitor targeting angiogenic kinases such as VEGFR, FGFR, and platelet-derived growth factor receptor (PDGFR) as well as mutant oncogenic kinases such as c-kit receptor tyrosine kinase (KIT), RET, and BRAF [67]. Several multicenter phase II trials were conducted to assess the efficacy of regorafenib for chemotherapy-refractory patients with BTC [68,69,70]. Reported results included ORRs of 0–11%, median PFS of 3.0–3.9 months, and median OS of 5.3–7.9 months. One study found that elevated baseline levels of vascular endothelial growth factor D were associated with poor PFS, while elevated baseline levels of interleukin 6 and glycoprotein 130 were associated with poor OS [70]. Commonly observed severe AEs were hypophosphatemia, hyperbilirubinemia, hypertension, hyponatremia, and hand-foot skin reaction.

The efficacy of regorafenib in combination with avelumab, a PD-L1 inhibitor, was also investigated in a phase II trial for BTC patients refractory to prior chemotherapy [71]. The trial reported an ORR of 13.8%, median PFS of 2.5 months, and median OS of 11.9 months. Higher baseline levels of PD-L1 and indoleamine 2,3-dioxygenase expression were associated with better outcomes.

Lenvatinib is an oral multikinase inhibitor targeting VEGFR, FGFR, PDGFR, RET, and KIT. A multicenter phase II trial was conducted to evaluate the efficacy of lenvatinib as second-line treatment for patients with unresectable BTC. This trial showed an ORR of 11.5%, median PFS of 3.19 months, and median OS of 7.35 months [72]. Common severe AEs included hypertension, proteinuria, palmar-plantar erythrodysesthesia, decreased appetite, and anemia.

Combination therapy with lenvatinib and PD-1 inhibitors have also been investigated. On study enrolled 38 patients with advanced BTC to receive lenvatinib plus a PD-1 inhibitor (pembrolizumab/tislelizumab/sintilimab/camrelizumab/toripalimab) as a first-line treatment [73]. The ORR was 42.1%, the DCR was 76.3%, and the median OS was 17.7 months. Another study investigated lenvatinib plus pembrolizumab as a non-first-line treatment for patients with refractory advanced BTC [74]. The ORR was 25%, the DCR was 78.1%, median PFS was 4.9 months, and median OS was 11.0 months. Bilirubin elevation, hypertension, pneumonitis, and fatigue were reported as severe AEs.

### 3.8. Poly (Adenosine Diphosphate-Ribose) Polymerase (PARP) Inhibitors

*BRCA1/2* play a role in the DNA repair process via the homologous recombination repair pathway. Other components of the DNA repair pathway include *MSH6*, *BAP1*, *ATM*, *MLH1*, and *MSH2* [41]. *BRCA1/2* mutations are detected in 1–7% of patients with BTC, and a broader spectrum of DNA damage repair gene alterations is identified in 28.9–63.5% of patients with BTC [75,76,77]. While homologous recombination deficiency (HRD) promotes tumorigenesis, malignancies of patients with HRD are sensitive to platinum-based chemotherapy as well as HRD-targeted therapy, such as PARP inhibitors [77]. A phase II trial was conducted to evaluate the efficacy of the oral PARP inhibitor olaparib plus durvalumab in advanced solid tumors with HRD [78]. Forty-eight patients with HRD were enrolled, of which sixteen harbored *BRCA1/2* alterations. Two patients with BTC were included in the study; both had *BRCA1/2* alterations, and both achieved partial response.

### 3.9. Microsatellite Instability (MSI)-High/Tumor Mutational Burden (TMB)-High

Tumors exhibiting mismatch repair deficiency are notably prone to mutations in repetitive DNA sequences, leading to high MSI. In MSI-high tumors, programmed death ligand 1 (PD-L1) expression can lead to the suppression of adaptive immune responses [79]. Programmed death 1 (PD-1) functions as the receptor for PD-L1, which is expressed on T cells. Immunotherapy inhibits PD-1/PD-L1, enabling T cells to recognize and eliminate cancer cells. One report suggests that heterogeneity of T cells in GBC may influence immunotherapy strategies [73]. MSI-high and TMB-high were observed in 2.2–3.2% and 3.4% of BTC, respectively [80,81,82]. The efficacies of immunotherapy in conjunction with chemotherapy were reported [83].

Pembrolizumab is an intravenous monoclonal antibody binding to PD-1 expressed on lymphocyte and blocks binding to PD-L1. Pembrolizumab monotherapy was given to 233 MSI-high patients, including 22 BTC patients in a multicenter, phase II, basket trial (KEYNOTE-158) [84]. The ORR was 40.9%, median PFS was 4.2 months, and median OS was 24.3 months. Two patients with BTC achieved complete response. The results with pembrolizumab monotherapy in TMB-high patients (defined as patients with at least 10 mutations per megabase) were also evaluated [85]. Within the entire cohort, 13% had high TMB, and the ORR was 29% in TMB-high patients. However, BTC patients were not included in the above cohort.

Durvalumab is an intravenous PD-L1 inhibitor which also has reported efficacy in combination with GC. The TOPAZ-1 trial found greater efficacy with GC plus durvalumab than GC alone [8]. While only 0.7% of included patients were MSI-high, favorable efficacy was achieved with GC plus durvalumab, with OS of 12.8 vs. 11.5 months (hazard ratio: 0.80; *p* = 0.021) PFS of 7.2 vs. 5.7 months (hazard ratio: 0.75; *p* = 0.001). The ORR was also higher in patients treated with GC plus durvalumab (26.7% vs. 18.7%), showing additional benefit from durvalumab regardless of MSI status.

Although still in the animal study stage, the efficacy of combined therapy (anti-CTLA-4 inhibitor with anti-PD-1 inhibitor and GC) for ICC has been reported [86].

### 3.10. Bifunctional Antibody Therapy

Bintrafusp alfa is an intravenous bifunctional fusion protein comprising a PD-L1 monoclonal antibody fused with an extracellular domain of the human transforming growth factor beta (TGF-β) receptors, which functions as a trap for TGF-β within the tumor environment. A multicenter phase II trial was conducted to evaluate bintrafusp alfa as second-line treatment for patients with advanced BTC following first-line platinum-based chemotherapy [87]. Reported results included an ORR of 10.7%, median PFS of 1.8 months, and median OS of 7.6 months. The observed severe AEs were anemia, pruritus, and increased alanine aminotransferase. This was a negative study, with the primary endpoint (ORR) not being achieved.

The common mutations observed in BTC were shown in Table 1 by primary site, and phase II and phase III trials are summarized in Table 2.

## 4. Ongoing Clinical Trials

Currently ongoing clinical trials are shown in Table 3. There are many clinical trials targeting FGFR. For first-line treatment, there are three ongoing phase III trials comparing the FGFR inhibitor with GC. For refractory cases, there are several clinical trials evaluating new FGFR inhibitors. Phase II trials with PARP inhibitors such as olaparib and nirapalib are being conducted for patients with HRD or *IDH* mutations. Clinical trials using multikinase inhibitors in combination with chemotherapy are conducted mainly in China. A phase II/III trial of paclitaxel plus CTX-009, a bispecific antibody which simultaneously inhibits Delta-like ligand 4/Notch and vascular endothelial growth factor A, is also being conducted.

In recent years, techniques to generate BTC patient-derived organoids had been established. The success rate of generating BTC patient-derived organoids were reported to be 74.4–88.2% [88,89]. This technique has the potential to solve the difficulty of collecting sufficient quantities of tumor cells for genetic analysis in BTC patients and can make precision medicine more feasible.

## 5. Conclusions

Although available chemotherapy for BTC is limited, treatment options are gradually increasing due to advances in precision medicine. Many challenges remain, including the collection of sufficient tissue to identify targetable genetic alterations and difficulty in detecting fusion genes through liquid biopsies. However, the development of novel techniques, such as organoids, may help overcome the challenges. Systems need to be put in place for more patients to receive genetic analysis. The detection of selected targetable genetic alterations has led to effective treatment in an increasing number of cases, showing promise for future drug development and improvement of prognosis.

## Figures and Tables

**Table 1:** Common mutation by primary site.

Primary Site	Genetic Alterations	Rate
iCCA	FGFR2	7–14%
	IDH1/2	20–29%
	BRAF	3–7%
pCCA and dCCA	KRAS	38–57%
GBC	ERBB2	6–15%
all BTC	RET	0–5%
	NTRK	0.2–0.7%
	MSI-high	2.2–3.2%

iCCA, intrahepatic cholangiocarcinoma; pCCA, perihilar cholangiocarcinoma; dCCA, distal cholangiocarcinoma; GBC, gallbladder cancer; BTC, biliary tract cancer; FGFR, fibroblast growth factor receptor; IDH, isocitrate dehydrogenase; RET, rearranged during transfection; MSI, microsatellite instability.

**Table 2:** Clinical trials results.

Target	Drug	Phase	Sample Size	Treatment Status	ORR	Median PFS (Months)	Median OS (Months)
FGFR	Pemigatinib [23]	II	146	Refractory	35.5%	6.9	21.1
	Infigratinib [25]	II	108	Refractory	23.1%	7.3	12.2
	Futibatinib [28]	II	103	Refractory	41.7%	9.0	17.1
IDH	Ivosidenib [39]	III	185	Refractory	2.4%	2.7	10.3
KRAS	Adagrasib [42]	II	12	Refractory	41.7%	8.6	15.1
BRAF/MEK	Dabrafenib/Trametinib [46]	II	43	Refractory	47%	9	14
HER2	Pertuzumab/Trastuzumab [51]	II	39	Refractory	23.1%	4.0	10.9
	Trastuzumab deruxtecan [53,54]	II	22/41	Refractory	22.0–36.4%	4.4–4.6	7–7.1
	Tucatinib/Trastuzumab [55]	II	30	Refractory	46.7%	5.5	15.5
	Trastuzumab/FOLFOX [56]	II	34	Refractory	29.4%	5.1	10.7
	Trastuzumab/GC [57]	II	90	Naïve	55.5%	7	9.96
	Zanidatamab [58]	II	87	Refractory	41.3%	5.5	immature
	Neratinib [59]	II	25	Refractory	16%	2.8	5.4
Multikinase	Regorafenib [68,69,70]	II	39/43/66	Refractory	0–11%	3.0–3.9	5.3–7.9
	Regorafenib/Avelumab [71]	II	34	Refractory	13.8%	2.5	11.9
	Lenvatinib [72]	II	26	Refractory	11.5%	3.19	7.35
	Lenvatinib/PD-1 inhibitor [73]	II	38	Naïve	42.1%	4.9	11
PARP	Olaparib/Durvalumab [78]	II	2	Refractory	100%	no data	no data
MSI-high	Pembrolizumab [84]	II	22	Refractory	40.9%	4.2	24.3
Bifunctional antibody	Bintrafusp alfa [87]	II	159	Refractory	10.7%	1.8	7.6

ORR, overall response rate; PFS, progression free survival; OS, overall survival; FGFR, fibroblast growth factor receptor; IDH, isocitrate dehydrogenase; HER2, human epidermal growth factor receptor 2; FOLFOX, fluorouracil and leucovorin plus oxaliplatin; GC, gemcitabine plus cisplatin; MSI, microsatellite instability; PD-1, programmed death 1; PARP, poly (adenosine diphosphate-ribose) polymerase.

**Table 3:** Ongoing clinical trials.

Target	Drug	Phase	Planned Sample Size	Treatment Status	Study Number
FGFR	Pemigatinib (vs. GC)	III	434	Naïve	NCT03656536
	Infigratinib (vs. GC)	III	350	Naïve	NCT03773302
	Futibatinib (vs. GC)	III	216	Naïve	NCT04093362
	Pemigatinib + PD-1 inhibitor	II	30	Naïve	NCT05913661
	Tinengotinib (vs. Chemotherapy)	III	200	Refractory	NCT05948475
	Derazatinib + Atezolizumab	II	37	Refractory	NCT05174650
	Futibatinib	II	120	Refractory	NCT05727176
	Gunagratinib	II	64	Refractory	NCT05678270
	HMPL-453 tartrate	II	128	Refractory	NCT04353375
	Tasurgratinib	II	60	Refractory	NCT04238715
IDH	Ivosidenib	IIIb	220	Refractory	NCT05876754
	Olaparib	II	145 *	Refractory	NCT03212274
	Olaparib + Durvalumab	II	58 *	Refractory	NCT03991832
	Olaparib + Ceralasertib	II	50 *	Refractory	NCT03878095
RAS-BRAF-MEK -ERK pathway	mFOLFOX ± Binimetib	II	66	Refractory	NCT05564403
	Trametinib + Hydroxychloroquine	II	30	Refractory	NCT04566133
HER2	Distamab Vedotin + Zimberelizumab	II	31	Refractory	NCT05540483
	Zanidatamab + Chemotherapy	II	362 *	Naïve	NCT03929666
	Trastuzumab + Pertuzumab	II/III	30 *	-	NCT05786716
	Trastuzumab + modified FOLFOX-6	II	36	Refractory	NCT04722133
	Trastuzumab + Tucatinib	II	270 *	Refractory	NCT04579380
NTRK	Larotrectinib	II	204 *	Refractory	NCT02576431
PARP	Olaparib ± Durvalumab	II	62	Maintenance	NCT05222971
	AZD6738 + Olaparib or AZD6738 + Durvalumab	II	74	Refractory	NCT04298021
	Olaparib	II	36	After platinum-based Chemotherapy	NCT04042831
	Niraparib + Dostarlimab	II	112 *	Refractory	NCT04779151
Multikinase	Lenvatinib + Tislelizumab + XELOX	II	20	Naïve	NCT05291052
	Tislelizumab + GEMOX ± Lenvatinib	II	60	Naïve	NCT05620498
	GC ± Lenvatinib and Tislelizumab	II	100	Naïve	NCT05532059
	Lenvatinib + Tislelizumab + GC (vs. GC)	III	80	Naïve	NCT05823311
	Lenvatinib + Durvalumab ± Chemotherapy	II	40	Naïve	NCT05935579
	Lenvatinib + Envofolimab + GC	II	43	Naïve	NCT05410197
	Lenvatinib + Pembrolizumab	II	40	Refractory	NCT04550624
	Lenvatinib + Toripalimab	II	44	Refractory	NCT04211168
	Lenvatinib + Paclitaxel	II	55	Refractory	NCT05170438
	Surufatinib + Toripalimab	II	30	Refractory	NCT05056116
	Surufatinib + Cadonilimab	II	48	Refractory	NCT06092645
	Regorafenib + Cadonilimab + GC	II	30	Naïve	NCT05820906
	Anlotinib + TQB2450 + nab-paclitaxel + cisplatin	II	20	Naïve	NCT05812430
	Donafenib + Tislelizumab + GEMOX	II	35	Naïve	NCT05668884
	Donafenib + Sitilimab + HAIC	II	32	Naïve	NCT05348811
	Nilotinib + intravenous and intraperitoneal Paclitaxel	II	70 *	Refractory	NCT05185947
Bispecific antibody	CTX-009 + Paclitaxel	II/III	150	Refractory	NCT05506943

* Basket trial including cancers other than BTC. FGFR, fibroblast growth factor receptor; GC, gemcitabine plus cisplatin; PD-1, programmed death 1; IDH, isocitrate dehydrogenase; HER2, human epidermal growth factor receptor 2; FOLFOX, fluorouracil and leucovorin plus oxaliplatin; NTRK, neurotrophic tropomyosin receptor kinase; PARP, poly (adenosine diphosphate-ribose) polymerase; XELOX, capecitabine plus oxaliplatin; GEMOX, gemcitabine plus oxaliplatin; HAIC, hepatic arterial infusion chemotherapy.

## Abbreviations

BTC	biliary tract cancer
dCCA	distal cholangiocarcinoma
GBC	gallbladder cancer
iCCA	intrahepatic cholangiocarcinoma
pCCA	perihilar cholangiocarcinoma
FGFR	fibroblast growth factor receptor
ORR	overall response rate
PFS	progression free survival
OS	overall survival
DCR	disease control rate
AEs	adverse events
IDH	isocitrate dehydrogenase
2-HG	2-hydroxyglutarate
HER2	human epidermal growth factor receptor 2
T-Dxd	trastuzumab deruxtecan
IHC	immunohistochemistry
ILD	interstitial lung disease
NTRK	neurotrophic tropomyosin receptor kinase
MSI	microsatellite instability
TMB	tumor mutational burden;
PD-L1	programmed death ligand 1
PD-1	programmed death 1
GC	gemcitabine plus cisplatin
DDR	DNA damage response
RET	rearranged during transfection
VEGFR	vascular endothelial growth factor
PDGFR	platelet-derived growth factor receptor
KIT	c-kit receptor tyrosine kinase
PARP	poly (adenosine diphosphate-ribose) polymerase
HRD	homologous recombination deficiency
TGF-β	transforming growth factor beta
